# Risk stratification in myeloma by detection of circulating plasma cells prior to autologous stem cell transplantation in the novel agent era

**DOI:** 10.1038/bcj.2016.117

**Published:** 2016-12-16

**Authors:** R Chakraborty, E Muchtar, S K Kumar, D Jevremovic, F K Buadi, D Dingli, A Dispenzieri, S R Hayman, W J Hogan, P Kapoor, M Q Lacy, N Leung, M A Gertz

**Affiliations:** 1 Department of Internal Medicine, Division of Hematology, Mayo Clinic, Rochester, MN, USA; 2 Department of Internal Medicine, Hospitalist Services, Essentia Health-St Joseph's Medical Center, Brainerd, MN, USA; 3 Department of Laboratory Medicine and Pathology, Division of Hematopathology, Mayo Clinic, Rochester, MN, USA

## Abstract

The impact of circulating plasma cells (CPCs) prior to autologous stem cell transplantation (ASCT) for multiple myeloma has not been defined in the novel agent era. We evaluated the impact of pre-transplant CPCs, detected by six-color flow cytometry in patients undergoing early ASCT on post-transplant response, progression-free survival (PFS) and overall survival (OS). CPCs were detected in 162 out of 840 (19.3%) patients, with the median number of CPCs being 58 per 150 000 events. Ninety-nine percent of patients had received proteasome inhibitor and/or immunomodulator-based induction. The incidence of post-transplant stringent complete response (sCR) in the subgroups with and without CPCs was 15% and 38%, respectively, (*P*<0.001). The median PFS in the subgroups with and without CPCs was 15.1 (95% confidence interval (CI), 12.5–17.8) and 29.6 months (95% CI, 26.2–32.8), respectively, and the median OS was 41.0 months (95% CI, 32.6–58.2) and not reached (NR) (95% CI, 99.1-NR), respectively, (*P*<0.001 for both). On multivariate analysis for OS, factors independently predictive of mortality were the presence of CPCs (hazard ratio (HR) 2.5; 95% CI, 1.8–3.6; *P*<0.001) and sCR post transplant (HR 0.4; 95% CI, 0.2–0.6; *P*<0.001). Presence of CPCs prior to transplant has a high prognostic impact and should be prospectively validated in clinical trials.

## Introduction

The evolving paradigm of circulating plasma cells (CPCs) and its negative prognostic impact in plasma cell disorders has been demonstrated along the entire spectrum of the disease, from monoclonal gammopathy of undetermined significance to relapsed/refractory multiple myeloma (MM).^1, 2, 3, 4, 5, 6, 7, 8, 9, 10, 11^ Although the biology of CPCs is not well understood, immunophenotypic and functional characterization has revealed CPCs to be a highly clonogenic side population with functional resemblance to hematopoietic stem cells.^1^ CPCs have been shown to be quiescent, and hence, can be potentially overlooked by using only M-protein response assessment owing to the absence of monoclonal protein production.^1, 12, 13^

The therapeutic arena of MM has seen the introduction of new agents in the last decade, including proteasome inhibitors (PIs), immunomodulators (IMiDs), histone deacetylase inhibitors and monoclonal antibodies. However, patients with high-risk disease still experience poor outcomes, and have not been able to completely reap the benefits of novel agents and autologous stem cell transplantation (ASCT).^14^ The impact of the depth of response prior to transplant on survival in MM has been controversial, with conflicting evidence across various studies.^15, 16, 17, 18, 19, 20, 21^ In the era predating the widespread use of PIs and IMiDs, the presence of CPCs at transplant by immunohistochemistry or two-color flow cytometry was shown to predict a shorter disease-free survival and overall survival (OS).^22, 23^ Because of the ease of performing multiparameter flow cytometry (MFC) assays,^24, 25^ it has become easier to detect clonal CPCs in the peripheral blood with a high sensitivity.

Given the current state of knowledge regarding risk stratification prior to transplant in MM, some questions on CPCs still remain unanswered:^1^ do CPCs detected prior to transplant have any impact on transplant outcome in the era of novel agent-based induction and post-transplantation therapies?^2^ Does the identification of CPCs add further prognostic information to the current paradigm of risk stratification, including stage, high-risk cytogenetics and pre-transplant response by the current consensus response criteria?

In this retrospective study, we have investigated the incidence of CPCs prior to transplant using six-color MFC in the era of novel agents, and their impact on best post-transplant response, progression-free survival (PFS) and OS.

## Patients and methods

### Patients

This study was approved by the Mayo Clinic Institutional Review Board,and was conducted in accordance with federal regulations and the principles of the Declaration of Helsinki. Informed consent was obtained from all patients for review of their medical records. Routine six-color MFC for identification of CPCs was initiated at Mayo Clinic in January 2007 and is performed in all patients as a part of the pre-transplant evaluation. Testing for CPCs was carried out prior to stem cell mobilization. We have included all consecutive patients who underwent ASCT in our institution from January 2007 to May 2015, with an intent for early ASCT (within 12 months of diagnosis).

Mononuclear cells from peripheral blood samples were isolated by Ficoll gradient, and subsequently stained with antibodies to CD19, CD38, CD45, CD138, and cytoplasmic kappa and lambda immunoglobulin light chains.^24^ The CPCs were detected by the analysis of CD19, CD45, CD38 and CD138, and clonality was assessed by light chain restriction (kappa:lambda expression ratio of 4:1 (kappa restricted) or <1:2 (lambda restricted)). Flow cytometry was performed on BD FACSCanton II instruments (Becton Dickinson, Franklin Lakes, NJ, USA) and data were analyzed using BD FACSDiva software (Becton Dickinson). The target for collection was >150 000 cellular events. The clonal CPCs were reported as the number of clonal events/150 000 total collected events. For samples where <150 000 clonal events were examined, the number of final clonal events was adjusted to 150 000 events.

The primary end point of this study was post-transplant response, PFS and OS. Response was determined according to the current consensus response criteria.^26^ Best post-transplant response was defined as the best response at any time after ASCT in the first plateau. PFS was defined as time from transplant to disease progression or death due to any cause. OS was defined as time from transplant to death due to any cause. Patients who were alive and free of disease at the last follow-up visit were censored. High-risk cytogenetics by interphase fluorescence *in situ* hybridization (FISH) on bone marrow plasma cells (BMPCs) was defined by at least one of the following abnormalities: *t*(4;14), del(17p), *t*(14;16), *t*(14;20) or +1q^27, 28, 29^ detected at any time point from diagnosis to transplant.

Stem cell mobilization, conditioning and transplant management in Mayo Clinic has been previously described.^30, 31^ All mobilizations were performed with granulocyte colony-stimulating factor subcutaneously (10 mcg/kg per day). Granulocyte colony-stimulating factor was given alone or after cyclophosphamide (1.5 g/m^2^ daily for 2 consecutive days), or with plerixafor (routinely available since 2009). Full-dose melphalan (200 mg/m^2^) or reduced-dose melphalan (140 mg/m^2^) was given at physician discretion depending on general fitness, comorbidities and renal function.

### Statistical analysis

Two-sided Fisher's exact tests were used to test for differences between categorical variables. Two-sided Wilcoxon rank sum tests were used to compare continuous variables. Survival analysis was carried out using the method described by Kaplan and Meier.^32^ Differences in survival between groups were tested for statistical significance using the two-sided log-rank test. Univariate analysis using the Cox proportional hazards model was performed with the following variables: age ⩾65 years, HR cytogenetics, International staging system (ISS) stage 3, CPCs, very good partial response or better at transplant, stringent complete response (sCR) post transplant and melphalan dose. Factors prognostic for PFS and OS with a *P-*value <0.1 in the univariate analysis were studied in a multivariate analysis. JMP 10.0.0 (SAS Institute Inc., Cary, NC, USA) statistical package was used for all statistical analysis.

## Results

The study included a total of 840 patients who met the above-mentioned inclusion criteria. The median age of patients at transplant was 61 years (range, 24–76). Eight hundred and thirty (99%) patients had received novel agent-based induction therapy with PIs and/or IMiDs, including 277 (33%) patients who had received both PI and IMiD.

The median follow-up was 44 months from ASCT (95% confidence interval (CI), 40–47 months). Three hundred and twenty-one (38%) patients had received post-transplant maintenance or consolidation therapy. The median PFS of the entire cohort was 25.3 months (95% CI, 24.0–27.3). The median OS of the entire cohort was 100.0 months (95% CI, 90.7-not reached (NR)) and the 5-year-OS rate was 65% (95% CI, 61–70).

CPCs were detected on flow cytometry in 162 out of 840 (19.3%) patients prior to ASCT. The median number of CPCs among these 162 patients was 58 (range, 1–102 381) per 150 000 events collected. Data on cytogenetics by FISH were available for 742 out of 840 patients. Patients with CPCs had a higher incidence of harboring high-risk cytogenetics by FISH compared with those without CPCs (32.7% vs 20.4%, respectively; *P*=0.001). Among individual cytogenetic abnormalities, the frequency of *t*(4;14) was over twofold greater in patients with CPCs compared with those without (16.6% vs 7.3% *P*<0.001). The frequency of deletion 13q or monosomy 13 was also higher in the subgroup of patients with CPCs (55.6% vs 41.3% *P*=0.002). There was no significant difference in the frequency of deletion 17p, *t*(14;16), *t*(14;20) and +1q. The frequency of individual cytogenetic abnormalities in patients with and without CPCs is depicted in Table 1. On comparing the pre-transplant response, the incidence of very good partial response (VGPR) or better at transplant was lower in patients with CPCs compared with those without (22% vs 47%, respectively; *P*<0.001). Similarly, only 4.9% of patients with CPCs were in CR prior to transplant compared with 18.9% of patients without CPCs (*P*<0.001). The incidence of receiving induction therapy with a PI-based regimen was lower in patients with CPCs compared with those without (26% vs 38% *P*=0.004). There was no difference in the frequency of IMiD-based induction therapy in the two groups. The baseline characteristics of the entire cohort and the two subgroups are shown in Table 2.

A total of 283 (34%) patients had attained sCR after transplant. The incidence of sCR in the subgroups with and without CPCs was 15% and 38%, respectively, (*P*<0.001). The incidence of post-transplant CR (excluding sCR) in the respective subgroups was 13% and 15%, respectively, (*P=*0.502), and the incidence of post-transplant VGPR was 32% and 23%, respectively, (*P*=0.030).

**Figure 1:**
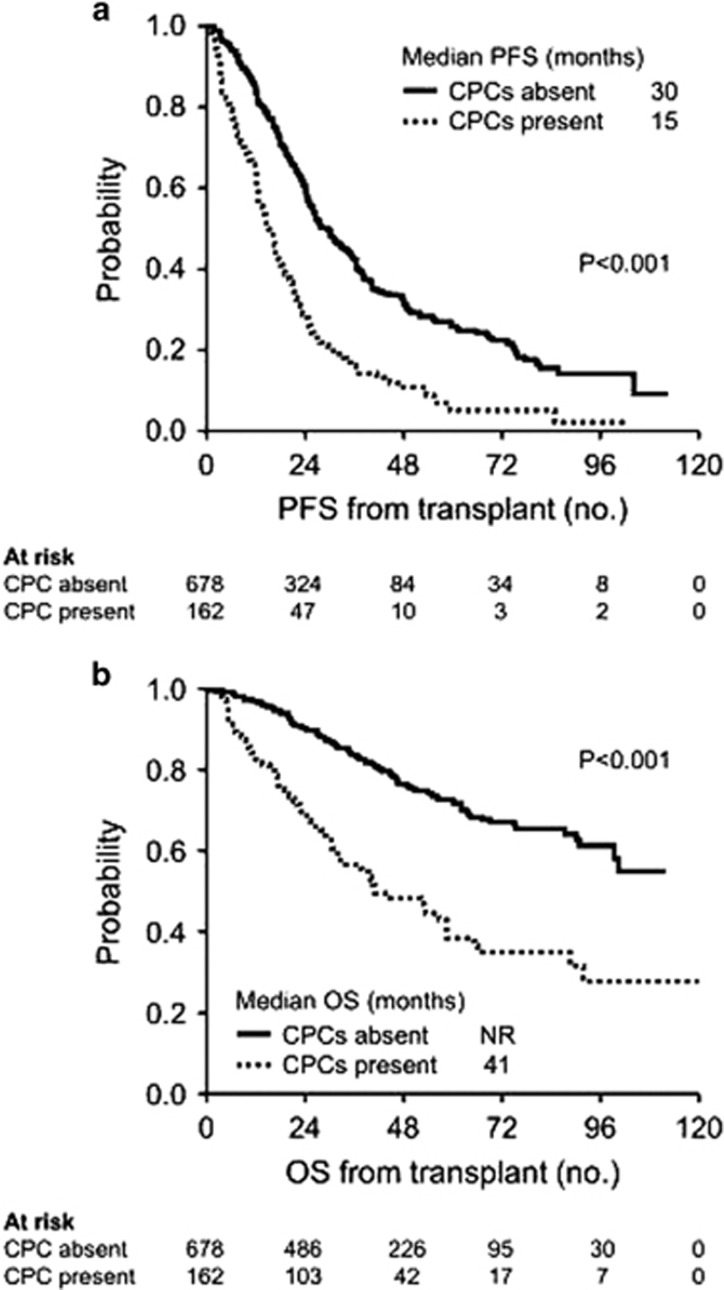
(**a**) Kaplan–Meier curves for PFS from transplant in patients with and without CPCs. (**b**) Kaplan–Meier curves for OS from transplant in patients with and without CPCs.

The median PFS in the subgroups with and without CPCs was 15.1 (95% CI, 12.5–17.8) and 29.6 months (95% CI, 26.2–32.8), respectively, and the median OS was 41.0 months (95% CI, 32.6–58.2) and NR (99.1-NR), respectively, (*P*<0.001 for both comparisons), with the 5-year-OS rates being 39% (95% CI, 30–49%) and 72% (95% CI, 68–77%), respectively. The Kaplan–Meier curves for PFS and OS is depicted in Figures 1a and b.

**Figure 2:**
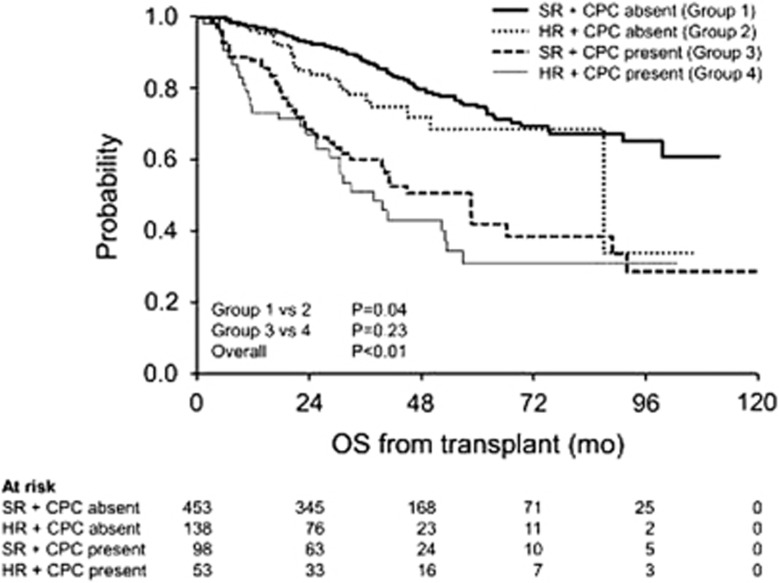
Kaplan–Meier curves for OS in patients stratified by the presence of CPCs and HR cytogenetics by FISH.

Subsequently, we investigated the median OS of patients stratified by the presence of CPCs and high-risk cytogenetics by FISH (Figure 2). In the subgroup without CPCs, the median OS of patients with high-risk (*n*=138) and standard-risk (*n*=453) cytogenetics was 86.8 months (95% CI, 86.8-NR) and NR (99.1-NR), respectively, (*P*=0.04). In the subgroup of patients with CPCs, the median OS of patients with HR (*n*=53) and standard risk (*n*=98) cytogenetics was 37.6 (95% CI, 25.3–53.0) and 58.2 months (32.5–88.3), respectively, (*P*=0.23).

In the univariate Cox model for PFS (Table 3), the following variables were associated with a significant impact on progression: high-risk FISH cytogenetics (hazard ratio (HR) 1.3; 95% CI, 1.1–1.6; *P*=0.015), presence of CPCs (HR 2.3; 95% CI, 1.9–2.8; *P*<0.001), ⩾very good partial response at transplant (HR 0.8; 95% CI, 0.7–0.9; *P*=0.012) and sCR post transplant (HR 0.5; 95% CI, 0.4–0.6; *P*<0.001). The factors which retained their significance on multivariate analysis included presence of CPCs (HR 2.0; 95% CI, 1.6–2.5; *P*<0.001) and sCR post transplant (HR 0.4; 95% CI, 0.3–0.6; *P*<0.001). In the univariate Cox model for OS (Table 3), the following variables were associated with a significant impact on mortality: high-risk FISH cytogenetics (HR 1.7; 95% CI, 1.2–2.3; *P*=0.001), presence of CPCs (HR 3.0; 95% CI, 2.2–3.9; *P*<0.001), sCR post transplant (HR 0.4; 95% CI, 0.3–0.6; *P*<0.001) and ISS stage 3 at diagnosis (HR 1.5; 95% CI, 1.1–2.0; *P*=0.015). The factors that retained their significance on multivariate analysis included presence of CPCs (HR 2.5; 95% CI, 1.8–3.6; *P*<0.001) and sCR post transplant (HR 0.4; 95% CI, 0.2–0.6; *P*<0.001).

## Discussion

Our study shows the negative prognostic impact of CPCs at transplant on post-transplant depth of response, PFS and OS, irrespective of the presence of high-risk cytogenetic abnormalities, stage at diagnosis and pre-transplant response in patients with newly diagnosed MM undergoing early ASCT. The prognostic impact of CPCs was so strong when present that FISH cytogenetics were no longer predictive of relapse-free or overall survival.

In a study on transplanted myeloma patients at Mayo Clinic who did not receive novel agent-based induction or maintenance, the median PFS and OS of patients with CPCs at transplant was 14 and 33 months, respectively,^23^ compared with 15 and 41 months, respectively, in our study in the era of PIs and IMiDs. However, their survival is still worse compared with patients with revised-ISS stage III, in whom the median PFS and OS from diagnosis are 29 and 43 months, respectively.^27^ As revised-ISS staging incorporates ISS stage, HR cytogenetics by FISH and serum lactate dehydrogenase, it is evident that the impact of CPCs is critical and independently defines an ultra-high-risk group, which has a worse survival irrespective of cytogenetic risk status or ISS stage at diagnosis. A phase 2 study of PI-based induction followed by ASCT in patients with primary plasma cell leukemia (defined by the presence of CPCs >2 × 10^9^/l or⩾20% of blood leukocytes) showed a median OS of 36 months,^33^ indicating poor outcomes compared with patients with standard-risk MM despite novel agent and upfront ASCT. In newly diagnosed multiple myeloma patients, quantification of CPCs at diagnosis has been shown to be a powerful tool to define high-risk disease independent of ISS stage at diagnosis.^3^

Patients with CPCs had a 50% higher risk of harboring high-risk cytogenetics (performed on BMPCs) compared with those with no CPCs. Upon assessing the frequency of individual cytogenetic abnormalities, the frequency of *t*(4;14) was over twofold in those with CPCs. Another study on 757 patients with newly diagnosed MM had also shown a significantly higher incidence of *t*(4;14) in patients with CPCs compared with those without (32% vs 19%, respectively; *P*=0.038).^10^ In a study performing FISH on paired samples of BMPCs and extramedullary plasma cells, *t*(4;14) was more prevalent in the extramedullary compartment, whereas del13q14 and 14q32 was more prevalent in the BM. Of note, the presence of CPCs was predictive of poor OS independent of the presence of *t*(4;14) in our study, indicating that clonal CPCs might contribute to the unfavorable prognosis in this cytogenetic subgroup. As CPCs have been shown to be unique cytogenetic subclones of BMPCs,^1^ paired cytogenetic analysis of CPCs and BMPCs should be performed in large cohorts of patients to identify the driver cytogenetic abnormalities, leading to their chemoresistance and replicative immortality.

Immunophenotypic characterization by MFC in CPCs by Paiva *et al.* has shown downregulation of several surface markers, including integrins, adhesion molecules, CD28, CD38, CD138, CD81 and CD117, among others, as compared with paired BMPCs. Furthermore, CPCs were shown to be mostly quiescent, in the subG0-G1 phase of cell cycle compared with BMPCs, which had also been confirmed in a prior study.^12^ They had a threefold colony and 16-fold cluster formation potential compared with their counterparts in the marrow. On the basis of these unique characteristics of quiescence and high clonogenic potential, the authors contemplated the possibility of CPCs representing a cancer stem cell-like population in myeloma.^1^ These findings imply that monoclonal antibodies against surface markers like CD38 might not be effective against CPCs. Myeloma cells with decreased surface expression of CD138 has also been shown to exhibit reduced sensitivity to lenalidomide.^34^

Myeloma with CPCs has also been proposed as a model for invasion and metastasis owing to intriguing similarities with the epithelial–mesenchymal transition program in solid tumors.^2^ The BM endosteal niche is quite hypoxic,^35^ which has been shown to induce quiescence, downregulate surface adhesion molecules and activate transcription factors in myeloma cells.^2, 36, 37^ BM hypoxia promotes dissemination of myeloma cells in the peripheral blood in a mouse model. It also leads to overexpression of CXCR4 in myeloma cells,^36^ which is known to have an important role in invasion and homing to new sites in solid tumors, leading to the metastatic cascade. CPCs in humans also overexpress CXCR4 compared with their bone marrow counterparts,^1^ which is critical in homing to new sites and heralding extramedullary disease.

Differences in DNA methylation patterns leading to epigenetic modulation of gene expression has been shown in both myeloma and plasma cell leukemia.^38, 39^ Furthermore, significantly increased hypermethylation has been observed in the subset of myeloma cells harboring *t*(4;14) translocation, which is more common in patients with CPCs compared with those without. The adenomatous polyposis coli gene, which functions as an antagonist of the Wnt signaling pathway, has been shown to be hypermethylated in the *t*(4;14) subset of myeloma cells.^38^ As Wnt pathway is known to be activated in both normal and cancer stem cell,^40^ it would be interesting to explore whether an activated Wnt pathway imparts stemness and self-renewal capacity in CPCs. In the hyperdiploid clones of myeloma cells, DNA methylation arrays have identified two distinct subgroups of patients with different methylation patterns. The subgroup with more heavily methylated genes had a significantly poorer OS compared with the other (45 versus 70 months; *P*=0.03), despite no difference in the incidence of known FISH-defined high-risk cytogenetic markers in the two groups.^38^

There are certain limitations of this study. First, this is a retrospective study and the findings need to be prospectively validated in clinical trials. Second, the quantification of CPCs are based on six-color MFC that is routinely performed in our institution. With the advent of more sensitive MFC (⩾eight-color) techniques, the frequency of detection of CPCs will increase and various cutoffs could be explored for further refinement of risk-stratification criteria. Nevertheless, this is the largest study showing the strong prognostic impact of CPCs prior to transplant in predicting survival independent of the currently employed risk-stratification systems in a homogeneously treated cohort of MM patients undergoing early ASCT. The proposed risk stratification with CPCs is easily reproducible and cost-effective. Detailed epigenomic, genomic and transcriptomic analysis of CPCs in large cohorts of patients is urgently needed to uncover the biological basis of their resistance to both high-dose cytotoxic therapy and novel agents, and identify potential avenues of targeted therapy against CPCs. Quantification of CPCs along the entire trajectory of the disease should be actively incorporated in clinical trials to study the clonal evolution and kinetics of CPCs, and its impact on disease outcomes.

## Figures and Tables

**Table 1:** Frequency of cytogenetic abnormalities by iFISH in different subgroups

*Cytogenetic abnormalities by iFISH (*N*=742)*	*CPCs absent (*n*=678)*	*CPCs present (*n*=162)*	P*-value*
Deletion 17p, *n* (%)	65 (11.0)	24 (15.9)	0.107
*t*(4;14), *n* (%)	**43 (7.3)**	**25 (16.6)**	**<0.001**
*t*(14;16), *n* (%)	22 (3.7)	6 (4.0)	0.883
*t*(14; 20), *n* (%)	6 (1.0)	2 (1.3)	0.747
+1q, *n* (%)	29 (4.9)	8 (5.3)	0.845
Deletion 13q or monosomy 13	**244 (41.3)**	**84 (55.6)**	**0.002**

Abbreviations: CPC, circulating plasma cell; iFISH, interphase fluorescence *in situ* hybridization; *N*, number of patients with available data on iFISH cytogenetics. Bold values indicate statistically significance parameters.

**Table 2:** Baseline clinical characteristics

*Baseline characteristics*	*Overall (*n*=840)*	*CPCs absent (*n*=678)*	*CPCs present (*n*=162)*	P*-value*
Median age at transplant (range), years	61.1 (24.4–76.1)	61.4 (24.4–76.1)	59.9 (32.2–73.8)	0.068
Males (%)	59.6	61.2	53.1	0.060
				
*ISS stage,* n *(%)*				*0.004*
Stage I	161 (19.2)	143 (21.1)	18 (11.1)	
Stage II	313 (37.3)	257 (37.9)	56 (34.6)	
Stage III	204 (24.3)	156 (23.0)	48 (29.6)	
Missing	162 (19.3)	122 (18.0)	40 (24.7)	
				
Median time from diagnosis to transplant (range), months	6.1 (1.3–11.9)	6.1 (1.3–11.9)	6.3 (2.4–11.9)	0.215
				
*FISH cytogenetics*^a^, n *(%)*				*0.001*
High risk	191 (22.7)	138 (20.4)	53 (32.7)	
Standard risk	551 (65.6)	453 (66.8)	98 (60.5)	
Missing	98 (11.7)	87 (12.8)	11 (6.8)	
				
*Pre-transplant response,* n *(%)*				*<0.001*
CR	136 (16.2)	128 (18.9)	8 (4.9)	
VGPR	221 (26.3)	193 (28.5)	28 (17.3)	
PR	346 (41.2)	284 (41.9)	62 (38.3)	
MR or SD	93 (11.1)	57 (8.4)	36 (22.2)	
PD	44 (5.2)	16 (2.4)	28 (17.3)	
				
*Induction regimen*				
PI-based only, *n* (%)	297 (35.4)	255 (37.6)	42 (25.9)	0.004
IMiD-based only, *n* (%)	256 (30.5)	206 (30.4)	50 (30.9)	0.905
PI- and IMiD-based, *n* (%)	277 (33.0)	211 (31.1)	66 (40.7)	0.021
				
*Melphalan dose,* n *(%)*				*0.938*
Full	740 (88.1)	597 (88.1)	143 (88.3)	
Reduced	100 (11.9)	81 (11.9)	19 (11.7)	
				
Post-transplant maintenance or consolidation, *n* (%)	321 (38.2)	262 (38.6)	59 (36.4)	0.600

Abbreviations: CPC, circulating plasma cell; CR, complete response; FISH, fluorescence *in situ* hybridization; IMiD, immunomodulator; ISS, International staging system; MR, minimal response; PD, progressive disease; PI, proteasome inhibitor; PR, partial response; SD, stable disease; VGPR, very good partial response.

a High-risk FISH cytogenetics was defined by *t*(4;14), del(17p), *t*(14;16), *t*(14;20) and +1q.

**Table 3:** Univariate and multivariate analysis for PFS and OS by Cox proportional hazards model

*Variable*	*Progression-free survival (PFS)*				*Overall survival (OS)*			
	*Univariate*	P*-value*	*Multivariate*	P*-value*	*Univariate*	P*-value*	*Multivariate*	P*-value*
	*Hazard ratio (95% CI)*		*Hazard ratio (95% CI)*		*Hazard ratio (95% CI)*		*Hazard ratio (95% CI)*	
Age ⩾65	0.87 (0.72–1.05)	0.144	NA	NA	1.09 (0.82–1.44)	0.538	NA	NA
HR FISH cytogenetics	1.30 (1.05–1.60)	0.015	1.20 (0.97–1.48)	0.088	1.71 (1.24–2.32)	0.001	1.26 (0.86–1.81)	0.231
CPCs present	2.28 (1.87–2.76)	<0.0001	**2.03 (1.64–2.50)**	**<0.001**	2.97 (2.25–3.88)	<0.001	**2.52 (1.78–3.55)**	**<0.001**
⩾VGPR at transplant	0.80 (0.67–0.95)	0.012	1.15 (0.92–1.42)	0.209	0.95 (0.72–1.24)	0.727	NA	NA
sCR post transplant	0.45 (0.37–0.55)	<0.001	**0.44 (0.34–0.55)**	**<0.001**	0.42 (0.30–0.59)	<0.001	**0.39 (0.25–0.61)**	**<0.001**
ISS stage 3	1.15 (0.94–1.41)	0.168	NA	NA	1.48 (1.08–2.01)	0.015	1.21 (0.86–1.70)	0.270
Reduced-dose melphalan	1.03 (0.78–1.32)	0.829	NA	NA	1.40 (0.96–1.99)	0.076	1.27 (0.78–1.98)	0.322

Abbreviations: CI, confidence interval; CPC, circulating plasma cells; FISH, fluorescence *in situ* hybridization; HR, high risk; ISS, International staging system; NA, not applicable; sCR, stringent complete response; VGPR, very good partial response. Bold values indicate statistically significance parameters.
